# Supplementary material for: Male circumcision uptake during the Botswana Combination Prevention Project
Source: PLoS One. 2022 Jun 15;17(6):e0269178. doi: 10.1371/journal.pone.0269178 (PMC9200323; doi:10.1371/journal.pone.0269178)
Supplement: Attachment — Submitted filename: Male circumcision uptake during the Botswana Combination Prevention Project.docx [file pone.0269178.s001.docx]

Male circumcision uptake during the Botswana Combination Prevention Project- PONE-D-21-21032

Abstract

This article’s main objective was to examine sociodemographic characteristics and referral procedures associated with VMMC uptake in the Botswana Combination Prevention Project (BCPP) and examined the effectiveness of referral of men to MC services from HIV testing venues. The article is more relevant to the setting of Botswana, where HIV/AIDS is still a concern. The abstract is well written and covers in a nutshell the key findings of the study.

**Introduction**

The authors have succinctly provided a background of the SMC programme in Botswana and have stated the gap which their study seeks to address. They have also provided the rationale for their study. Meanwhile my suggestion is that they should provide a more detailed explanation how their study differs with many previous studies on SMC that have been done in Botswana. What more information would the study add?

**Methodology**

Although the methodology of the study is well explained there are some few things to note. It is not clear how you measured the outcome and explanatory variables. That information is vital for making the reader to understand how variables were conceptualised and measured.

**Results**

Please kindly revise the interpretation of table 1. Table 1 presents the characteristics of men aged 16-49 already circumcised vs not circumcised at baseline, but the authors interpret the results of the table as if the results are for the multivariate models. These are just proportions and should not be interpreted using words such as more or less likely, to imply multivariate associations. As a result, the authors may need to revisit the interpretation of this table and use proper language. In the last sentence of this paragraph you refer to multivariate analysis, which table? Please consider ordering the results in a more logical way. Referring to multivariate when interpreting bivariate tables does not seem well.

**Discussion**

This section is written well but can be improved. The key finding of this study is that **Younger age, being unemployed, and undergoing HIV testing at mobile venues rather than at home were associated with current VMMC uptake.** This is not mentioned in the discussion section; my view is that the discussion should centre on the key findings of the study.
