# Supplementary material for: Male circumcision uptake during the Botswana Combination Prevention Project
Source: PLoS One. 2022 Jun 15;17(6):e0269178. doi: 10.1371/journal.pone.0269178 (PMC9200323; doi:10.1371/journal.pone.0269178)
Supplement: Attachment — Submitted filename: Response to reviewers.docx [file pone.0269178.s002.docx]

Tafireyi Marukutira

Burnet Institute

85 Commercial Road

Melbourne, 3004

Australia

Telephone: +61 422 161 316

Email: [tafireyi.marukutira@burnet.edu.au](mailto:tafireyi.marukutira@burnet.edu.au)

January 23, 2022

Editor-in-Chief

*PLOS ONE*

Dear Editors

Thank you for the opportunity to review our submitted manuscript, PONE-D-21-21032: “Male circumcision uptake during the Botswana Combination Prevention Project”. We feel the comments have improved the manuscript and hopefully have been fully addressed below. See below for a detailed response to reviewers’ comments. Please do not hesitate to contact us with any other concerns or questions.

The project has been supported by the President’s Emergency Plan for AIDS Relief (PEPFAR) through the Centers for Disease Control and Prevention (CDC) under the terms of Cooperative Agreements U2G GH000073 and U2G GH000419. The funding statement was removed in the manuscript. Please substitute this Role of Funder statement: “The funders, the U.S. CDC, designed the study, collected the data, and led the data analysis.”

Thank you for your consideration!

Sincerely,

Dr. Tafireyi Marukutira, PhD, MBChB, MPH

**RESPONSE TO REVIEWERS**

**Journal requirements:**

**1. When submitting your revision, we need you to address these additional requirements.**

**Please ensure that your manuscript meets PLOS ONE's style requirements, including those for file naming. The PLOS ONE style templates can be found at**

<https://journals.plos.org/plosone/s/file?id=wjVg/PLOSOne_formatting_sample_main_body.pdf> and

<https://journals.plos.org/plosone/s/file?id=ba62/PLOSOne_formatting_sample_title_authors_affiliations.pdf>

**Response**: Thank you and noted.

**2. Please provide additional details regarding participant consent. In the ethics statement in the Methods and online submission information, please ensure that you have specified whether consent was written or verbal/oral. If consent was verbal/oral, please specify: 1) whether the ethics committee approved the verbal/oral consent procedure, 2) why written consent could not be obtained, and 3) how verbal/oral consent was recorded**.

**Response:** Thank you. A written consent was required. This sentence was added in the ethics statement: “The VMMC procedure was conducted according to the standards for routine VMMC service delivery in Botswana and the WHO minimum package of services, including written informed consent, sexually transmitted infections screening and treatment, condom education and provision, and postoperative follow-up for documenting healing. Minors (≤18 years) required parental/guardian written consent and client assent.”

The online submission information was updated.

**3. You indicated that you had ethical approval for your study. In your Methods section, please ensure you have also stated whether you obtained consent from parents or guardians of the minors included in the study or whether the research ethics committee or IRB specifically waived the need for their consent.**

**Response**: Thank you, the following statement was added: “The VMMC procedure was conducted according to the standards for routine VMMC service delivery in Botswana and the WHO minimum package of services, including written informed consent, sexually transmitted infections screening and treatment, condom education and provision, and postoperative follow-up for documenting healing. Minors (≤18 years) required parental/guardian written consent and client assent.”

**Furthermore, please ensure you have included the registration number for the clinical trial referenced in the manuscript.**

**Response**: The protocol was registered on ClinicalTrials.gov as NCT01965470. This has been added in the Study Design subsection of the Methodology section.

**4. Thank you for stating the following in the Acknowledgments: Disclaimer Section of your manuscript:**

**“We would like to thank the study participants, and VMMC and HIV testing and counseling teams who made the Botswana Combination Prevention Project possible.**

**Disclaimer: This project has been supported by the President’s Emergency Plan for AIDS Relief (PEPFAR) through the Centers for Disease Control and Prevention (CDC) under the terms of Cooperative Agreements U2G GH000073 and U2G GH000419. The findings and conclusions in this report are those of the authors and do not necessarily represent the official position of the funding agencies. Preliminary results were presented at the International AIDS Society Conference (AIDS2018), Abstract #WEPEC235, Amsterdam, Netherlands, 23-27 July 2018.”**

**We note that you have provided funding information that is not currently declared in your Funding Statement. However, funding information should not appear in the Acknowledgments section or other areas of your manuscript. We will only publish funding information present in the Funding Statement section of the online submission form.**

**Please remove any funding-related text from the manuscript and let us know how you would like to update your Funding Statement. Currently, your Funding Statement reads as follows:**

**“This project has been supported by the President’s Emergency Plan for AIDS Relief (PEPFAR) through the Centers for Disease Control and Prevention (CDC) under the terms of Cooperative Agreements U2G GH000073 and U2G GH000419.”**

**Please include your amended statements within your cover letter; we will change the online submission form on your behalf.**

**Response:** The funding information in the “disclaimer” has been removed as requested. The funding statement described above is correct. Thank you.

**5. Thank you for stating the following financial disclosure:**

**“This project has been supported by the President’s Emergency Plan for AIDS Relief (PEPFAR) through the Centers for Disease Control and Prevention (CDC) under the terms of Cooperative Agreements U2G GH000073 and U2G GH000419.”**

**Please state what role the funders took in the study. If the funders had no role, please state: ""The funders had no role in study design, data collection and analysis, decision to publish, or preparation of the manuscript.""**

**If this statement is not correct you must amend it as needed.**

**Please include this amended Role of Funder statement in your cover letter; we will change the online submission form on your behalf.**

**Response:** The funding statement was removed in the manuscript. Please substitute this Role of Funder statement: “The funders, the U.S. CDC, designed the study, collected the data, and led the data analysis.”

**Reviewer #1: General comments:**

**The study design and analytic methods are appropriate and performed at a high standard in this manuscript. The only comments I had were requests to add in a little more information on some of the methods used, especially adding in citations.**

**Specific comments:**

**1. (line 93) How were the 30 communities selected? Were these randomly selected from a wider pool of communities or were these purposely selected? Furthermore, how were the 15 selected from the 30?**

**Response:** Thank you for highlighting this. The following statements were added: “The participating 30 communities were purposively selected based on desired size and accessibility. Matched-pair randomization was used to ensure that control and intervention groups were balanced on community size, baseline access to health services including ART, age structure, and geographic location.”

**2. (line 128) Please include the version and citation for Epi Info.**

**Response:** Thank you. Version and citation were added.

**3. (line 133) Please provide a citation for the Rao-Scott Chi-Squared test.**

**Response:** Thank you, citation was added.

**4. (lines 136-137) Since there are many different ways to account for clustering in logistic regression models, I strongly suggest you provide just a little more information on the method used here. In SAS, this could be done with GENMOD, GLIMMIX, SURVEYLOGISTIC, and other procedures. The SAS documentation should have the proper methodological citation.**

**Response**: PROC SURVEYLOGISTIC in SAS was used to account for clustering at the community-level. This has been added in the manuscript.

Reviewer #2:

**The authors use descriptive quantitative methods to describe male participants’ characteristics by baseline circumcision status and circumcision uptake among those uncircumcised at baseline, including among uncircumcised men referred from HIV testing services. Data are from a pair-matched community randomized HIV prevention trial to evaluate the effect of a combination prevention intervention on HIV incidence in Botswana. The analysis uses data from 15 intervention communities where VMMC data was collected. Analyzed data were collected between October 2013 and February 2016. Study findings indicate low rates of male circumcision uptake. Males who were younger, unemployed, or referred from a mobile HIV testing site were more likely to undergo circumcision. Reasons for declining circumcision included not being ready and fear of pain.**

**Introduction**

**1. A clear identification of the problem and strong rationale for the paper is not provided. For example, 22 million circumcisions in 10 years seems laudable. Why should we be concerned about slow uptake with what appears to be a high number of men circumcised?**

**Response**: Thank you for the comment. The introduction has been edited to address the gap.

**2. In concert with identification of the research problem, a review of studies that have examined or addressed the problem would strengthen the paper.**

**Response**: Thank you for the comment. The introduction has been edited to address the gap.

3. **Background information about male circumcision in Botswana (traditional and medical) and, if available, the study regions would also strengthen the introduction** and help the reader better understand the study and cultural context. For example, **what was the prevalence of male circumcision in Botswana at the time of the study**? **Was traditional circumcision being practiced**? **When was medical male circumcision for HIV prevention introduced in Botswana**? Was there a **target goal for proportion of males circumcised medically**? **Is Botswana one of the countries with slow uptake? If so, how does uptake in Botswana compare to the other priority countries**?

**Response**: Thank you for the comment. The introduction has been edited to address these gaps.

**4. How do the authors define “demand creation activities” and how do/can HIV testing services fit within demand creation activities?**

**Response**: Thank you for the comment. While counselling, education, media campaigns and financial incentives can be used for demand creation, counselling following HIV testing can also be used as an opportunity to offer VMMC to males with a negative result. This has been added to the introduction.

**5. Regarding lines 73-75, rather than presenting demand creation activities in general, it would be helpful to have a brief description of each activity listed (interpersonal mobilization, mass media, targeted service delivery).**

**Response**: Thank you for the comment. The introduction has been edited to address the gap.

**6. The purpose of the paper is not clearly stated. Is there a gap in knowledge that the authors seek to address? If so, what is the gap? Or is the intent to inform policy? Is it both?**

**Response**: Thank you for the comment. The introduction has been edited to address the gap. The following statement was edited as: “Here, we examine the uptake of VMMC among uncircumcised men through this pathway in intervention communities, and the demographic characteristics predicting obtaining circumcision in this way, as well as those associated with circumcision at baseline.

**Methods**

**1. Is the study a secondary data analysis or were the authors involved in BCPP? If the former, this should be stated.**

**Response**: The authors were involved in BCPP and the sub-analysis included intervention communities only, because of data quality issues in the standard of care arm.

**2. Line 96-97: A limitation of the study is that the data are old, and findings may not be applicable in the current Botswana context.**

**Response**: The discussion statement was added to address this potential limitation: “The findings remain relevant: Botswana missed the 2021 90% VMMC coverage target for 10-29 year olds, and there is no obvious reason why referral through HIV testing would be a less promising route to VMMC now than previously. However, it cannot be assumed that demographic associations have remained stable.”

3. Line 101: Unclear what is meant by “…follow-up for a 6-8 week period to cover the remaining 80% of the community residents.” Please clarify.

**Response**: Thank you. The sentence was edited to: “The testing campaign was conducted for a 6-8 week period to ensure that the remaining 80% of the community households were enumerated and offered HIV testing.”

**4. Lines 129-130: There is concern whether unique national identification numbers that were used to identify and link individuals across databases are available in the publicly available datasets. If they are, there is a serious risk of breach of confidentiality.**

**Response:** Thank you for highlighting this. The data for public release has been deidentified and the national unique identifiers are not in the data for public release. A new study identifier was assigned to protect the confidentiality of participants in the public release data. These statements were added in the manuscript.

**Results**

**1. The different ways males were informed about VMMC and whether they underwent circumcision is confusing. Please clarify.**

**Response**: Thank you. The introductory statement has been added to clarify circumcision status: “Of the 12,864 men identified through BCPP, 50% (n=6,448) reported already being circumcised at the time of intake. Among the 6,416 men not circumcised at baseline, 10% (n=635) underwent VMMC as part of study intervention during the 6-8 weeks of the HIV testing campaign.”

**Discussion**

**1. A key limitation of the data is that they are relative dated. It is likely that VMMC uptake has changed in important ways since the data were collected and therefore it is unclear that these data provide useful information for the current context. Some discussion around t**his limitation is needed.

**Response**: The discussion statement was added to address this potential limitation: “The findings remain relevant: Botswana missed the 2021 90% VMMC coverage target for 10-29 year olds, and there is no obvious reason why referral through HIV testing would be a less promising route to VMMC now than previously. However, it cannot be assumed that demographic associations have remained stable.”

**2. Missing is a discussion concerning the meaning and importance of the key findings that younger age, being unemployed, and undergoing HIV testing at mobile venues were associated with VMMC uptake. Further, how do these findings relate to those of other studies.**

**Response**: The relation of these findings with other studies is addressed in the second paragraph of the Discussion. Potential implications of these findings are now addressed there also.

**3. Line 213: What is the prevalence of traditional circumcision vs medical circumcision in Botswana?**

**Response**: Thank you. VMMC is the mainstay of Botswana’s program. This statement was added to the introduction: “Few tribal groups still offer traditional circumcisions at initiation ceremonies hence VMMC is the mainstay program in Botswana.”

**4. Line 217: What proportion of men were expected to be circumcised?**

**Response**: The scale-up of linkage to VMMC services targeted to ensure that >80% of HIV-negative men (aged 16–49) were circumcised. This has been added in the introduction.

**5. Earlier in the paper (see lines 94-95) the authors state that data on VMMC were only collected from the intervention communities. However, in lines 222-227, data on proportion of males circumcised in the standard of care arm are presented. Please clarify.**

**Response**: Thank you. As noted in the discussion, another BCPP analysis that used data from 20% of households was used for the overall evaluation of the trial. In contrast, here we present an analysis with data from all intervention communities. The referenced study was published in 2019 (N Engl J Med 2019;381:230-42. DOI: 10.1056/NEJMoa1812281)

**Reviewer #3:**

**Abstract**

**This article’s main objective was to examine sociodemographic characteristics and referral procedures associated with VMMC uptake in the Botswana Combination Prevention Project (BCPP) and examined the effectiveness of referral of men to MC services from HIV testing venues. The article is more relevant to the setting of Botswana, where HIV/AIDS is still a concern. The abstract is well written and covers in a nutshell the key findings of the study.**

**Introduction**

**The authors have succinctly provided a background of the SMC programme in Botswana and have stated the gap which their study seeks to address. They have also provided the rationale for their study. Meanwhile my suggestion is that they should provide a more detailed explanation how their study differs with many previous studies on SMC that have been done in Botswana. What more information would the study add?**

**Response**: Thank you. The introduction was edited to highlight the gap and the study aim.

**Methodology**

**Although the methodology of the study is well explained there are some few things to note. It is not clear how you measured the outcome and explanatory variables. That information is vital for making the reader to understand how variables were conceptualized and measured.**

**Response**: The “outcomes” subsection in the Methodology section has been made more explicit. The “data collection” section already states that the explanatory variables were obtained as self-reported values through home interviews but has been slightly edited to clarify this further. We will be happy to make any other needed clarifications if the reviewer could specify what is still unclear.

**Results**

**Please kindly revise the interpretation of table 1. Table 1 presents the characteristics of men aged 16-49 already circumcised vs not circumcised at baseline, but the authors interpret the results of the table as if the results are for the multivariate models. These are just proportions and should not be interpreted using words such as more or less likely, to imply multivariate associations. As a result, the authors may need to revisit the interpretation of this table and use proper language. In the last sentence of this paragraph you refer to multivariate analysis, which table? Please consider ordering the results in a more logical way. Referring to multivariate when interpreting bivariate tables does not seem well.**

**Response**: Thank you. The interpretation of Table 1 was revised to remove reference to “more likely” since this was a bivariate analysis. Multivariate analysis was not presented in a Table and was done for baseline and post-intervention covariates.

**Discussion**

**This section is written well but can be improved. The key finding of this study is that Younger age, being unemployed, and undergoing HIV testing at mobile venues rather than at home were associated with current VMMC uptake. This is not mentioned in the discussion section; my view is that the discussion should centre on the key findings of the study.**

**Response**: Thank you. An expansion of the second paragraph of the Discussion, discussing these findings, was added.
